# Hip Joint Synovial Cavity Thickness in Early Juvenile Idiopathic Arthritis Without Effusion: A Cross-Sectional Ultrasound Study

**DOI:** 10.3390/jcm15030962

**Published:** 2026-01-25

**Authors:** Zbigniew Żuber, Wojciech Kmiecik, Krzysztof Batko, Elżbieta Mężyk, Joanna Ożga, Magdalena Krajewska-Włodarczyk, Tomasz Madej, Bogdan Batko

**Affiliations:** 1 Department of Pediatrics, Faculty of Medicine and Health Sciences, Andrzej Frycz Modrzewski Krakow University, 30-705 Krakow, Poland; 2 Department of Dermatology, University Hospital, 31-503 Krakow, Poland; 3 Department of Rheumatology, School of Medicine, Collegium Medicum, University of Warmia and Mazury, 10-900 Olsztyn, Poland; m.krajewska-wlodarczyk@uwm.edu.pl; 4 Department of Pediatric Radiology, University Children’s Hospital, 20-093 Lublin, Poland; tomm74@poczta.onet.pl; 5 Department of Rheumatology and Immunology, Faculty of Medicine and Health Sciences, Andrzej Frycz Modrzewski University, 30-705 Krakow, Poland

**Keywords:** juvenile idiopathic arthritis, hip joint synovial cavity thickness, ultrasound, pediatric, reference

## Abstract

**Background**: The clinical meaning of hip joint synovial cavity thickness (HJSCT) on ultrasound (US) in juvenile idiopathic arthritis (JIA) without effusion is uncertain. **Methods**: In this cross-sectional study, we analyzed 369 children (187 JIA; 182 controls) undergoing hip US at a referral center in Kraków, Poland. JIA examinations were performed upon initial referral, early in the care pathway. We excluded patients with hip effusion and pre-existing inflammatory, traumatic or degenerative hip pathology. HJSCT was defined as the distance from the outer capsule margin to the femoral neck cortex. We used a Toshiba Aplio 400 system with a 12 MHz probe to measure and derive mean bilateral HJSCT. Bilateral concordance was assessed. Iterative multivariable linear regression modeling was used to compare groups, adjusting for non-linear age effects (natural splines) and WHO height-for-age z-scores (HAZ). **Results**: Left–right HJSCT agreement was high (ICC 0.947; mean difference 0.03 mm; 95% limits of agreement −0.64–0.70). In unadjusted analysis, mean (SD) HJSCT was similar in JIA versus controls: 5.83 (1.09) vs. 5.95 (0.99) mm, respectively (*p* = 0.25). In the final model (adj. R2 0.656), HJSCT was strongly associated with age (non-linear, *p* < 0.001) but not significantly associated with HAZ (β = 0.04; *p* = 0.11) or JIA status (β = 0.07; *p* = 0.30). Predicted HJSCT showed a steep increment in childhood and plateau in adolescence. **Conclusions**: In children without hip effusion, HJSCT mainly reflects physiological growth and does not differ significantly between early JIA patients and healthy controls. These findings suggest that capsular thickening is not a reliable standalone marker for early disease in the absence of effusion.

## 1. Introduction

Musculoskeletal disorders carry high socioeconomic and healthcare burden, with a range of geographic disparities [1]. Juvenile idiopathic arthritis (JIA) is a chronic immune-mediated disease that begins at an early age and impairs physical function [2,3]. It is also one of the most common rheumatic diseases in childhood, with pooled prevalence estimated at 70 cases per 100,000 persons in Europe [4]. Hip joint involvement is relatively common and estimated to occur in about 30–50% of JIA cases [5,6]. Even when the hip is uninvolved at diagnosis, it can often be affected later on during the course of JIA. Moreover, this may occur despite appropriate treatment [7]. In a cohort of newly diagnosed JIA patients, hip joint involvement changed from 6% to 19% within 5 years [7]. Recently, the ILAR [8] subtype classification was reappraised in a data-driven, pattern recognition approach that highlighted the importance of hip involvement as a poor prognostic factor [5]. Polyarticular and enthesitis-related subtypes are also more frequently tied to hip manifestations and have been associated with the general severity of joint arthritis [9]. While the knee, ankle, elbow and wrist are the most common joints affected upon first manifestation of JIA [7], all of these locations are easily accessible in physical examination. Meanwhile, reliable assessment of hip involvement is only possible through various imaging modalities. Early identification of transient or structural hip abnormalities remains of high clinical importance. It has been shown that hip involvement severity is tied to the burden of physical disability and is associated with arthritis duration [9]. The weight-bearing function of the hip joint is also critical in the adequate development of adult stature and physical fitness.

Despite years of research and emerging machine learning tools, there is no single biomarker or model that can predict JIA incidence or rate of progression [5,10]. JIA category alone is a poor predictor of short-term outcomes [11]. Prior studies have shown that age, gender, serostatus, microbiome, symmetry and extent of joint involvement, age at onset and disease duration affect prognosis in JIA, though these relationships are variable across studies [2,12,13,14]. Comprehensive analyses have shown that although numerous soluble molecules were investigated as prognostic/diagnostic aids, very few validation studies were conducted [15]. From a clinical standpoint, the current therapeutic approach remains focused on timely diagnosis and control of JIA activity using disease-modifying antirheumatic drugs. Other supportive treatment options are also emerging [16].

The optimal diagnostic approach for JIA is still not established, though recommendations are emerging [17,18,19]. Conventional radiography remains in use as a reference measure for peripheral joint destruction, with several validated scoring methods available [20]. Expert guidelines have also been published [21]. While US examination offers the promise of fast, noninvasive and low-cost imaging, no standardized approach has been agreed upon [22,23]. In part, this relates to operator dependency and limited validation of imaging findings that constrain widespread use and agreement. While US is increasingly used for peripheral joint evaluation in JIA, its application in hip imaging has largely focused on screening for joint effusion, synovitis or structural abnormalities often associated with established or later-stage disease [19,24]. In contrast, quantitative assessment of hip joint capsule measures in the absence of effusion (particularly in early-stage JIA) remains poorly described. The clinical significance of abnormalities in these measurements in early JIA has yet to be elucidated.

The frequent involvement of the hip in inflammatory conditions, coupled with the limited utility of physical examination and nonspecific nature of symptoms, makes this a location of high clinical importance (for an overview see Table 1). A retrospective study of 86 cases of pediatric post-infectious arthritis reported hip involvement in close to 50% of cases [25]. For JIA, hip abnormalities have been reported to affect between 30 and 50% of children [26]. US is viewed as a tool that should ideally exclude the presence of effusion [27]. Although the sensitivity for detecting effusion is reported to be over 90% in some studies [28], the type of fluid and temporal character (i.e., dynamics of the acute setting; sufficient time needs to pass for fluid to accumulate and become detectable) need to be considered [29]. However, aside from screening for effusion, US is also utilized to track physiological structural changes. Abnormalities in the rate or degree of growth may be relevant for differential of pathology.

Taken together, the aim of the present study was to describe hip joint synovial cavity thickness (HJSCT) in early JIA patients and healthy controls, and to evaluate its relationship with age, height, sex and disease status. By focusing on a population of patients without joint effusion in early JIA, the goal of this report is to characterize the inter-relationships between arthritis and anthropometric factors, which will extend the current evidence supporting application of reference charts for healthy children within the JIA population.

## 2. Materials and Methods

### 2.1. Study Overview

This observational cross-sectional study is based on US records of the pediatric hip joint among 369 children and adolescents. We extracted data on hip joint synovial cavity thickness (HJSCT) based on patient assessments at St. Ludwig’s Hospital, which is a reference pediatric rheumatology center in Kraków, Poland. We excluded patients with any of the following: (i) detectable joint effusion or (ii) history of pre-existing inflammatory, traumatic or degenerative hip pathology.

US examinations in the JIA patients were performed after referral to our center, early in the care pathway. However, due to inconsistent documentation, we were unable to reliably determine treatment naïvety or symptom duration prior to diagnosis; this limitation should be considered when interpreting the results.

Control participants were recruited from consecutive outpatients presenting for hip US imaging, without a final diagnosis of a specific disorder (the most frequent indication was exclusion of rheumatic disease, as per center specifics). Exclusion criteria in the control group were analogous, with the additional exclusion of growth abnormalities or relevant genetic conditions. Prior to study enrollment, we obtained written informed consent from parents or legal guardians. The study was granted approval by the local Bioethics Committee (Decision No. 99/KBL/OIL/2010).

### 2.2. Examination Protocol

Hip joint US was performed in all patients according to an internal protocol developed at our center (for a detailed description of our model approach, please see Appendix A.1). To reduce probe placement error, measurements of HJSCT were performed three times, bilaterally, with the recording of mean values as the final measure. The bilateral mean HJSCT was selected as the primary outcome to minimize random measurement variability and to provide a stable, bilaterally sensitive quantitative metric.

All examinations were conducted with a TOSHIBA Aplio 400 system with a 12 MHz linear transducer. There were three unblinded physicians who performed US assessments. Reproducibility of the adopted US approach to hip joint assessment was determined in a prior study; the intra- and inter-rater coefficients of variation were 0.5% and 12.4%, respectively [38].

**Figure 1:**
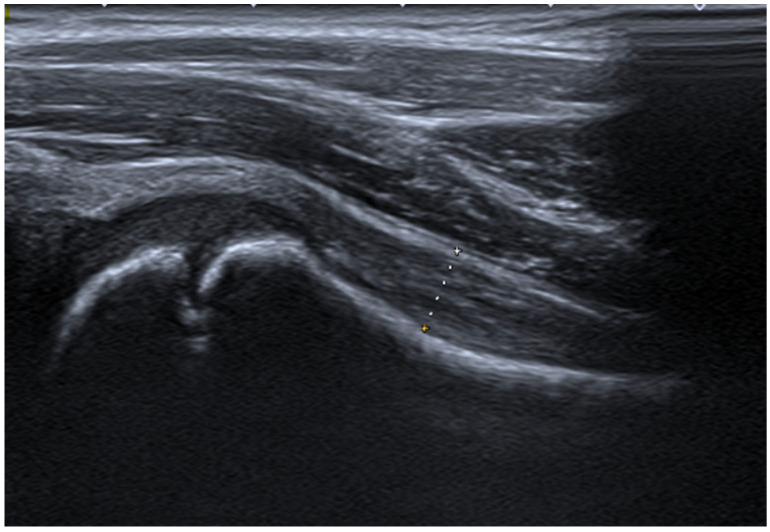
Sagittal imaging of the hip joint synovial cavity thickness in a 4-year-old patient using an anterior longitudinal sonographic scan.

US examination was performed in supine position, with the hip extended, in mild external rotation. First, the probe was placed in the sagittal position, parallel to the anatomical location of the femoral neck, at an angle of ~20° (see Figure 1). Assessments were performed following the anterior longitudinal approach, in which the HJSCT distance is measured as maximum of external joint capsule margin to the cortical edge of the femoral neck surface. Age-related reference values have been published previously by our team [38] and other groups [39].

### 2.3. Statistical Analysis

Analysis was performed in R version 4.5.2 (R Core Team, 2025; R Foundation for Statistical Computing, Vienna, Austria). Continuous variables were summarized using mean (standard deviation; SD) or median (interquartile range; IQR), as deemed appropriate. Categorical variables were reported as counts and percentages. Cross-group comparisons were reported using the Wilcoxon rank-sum test for continuous and chi-square test for categorical variables.

This exploratory study relied on a convenience sample of consecutive, eligible patients, with no a priori sample size calculation performed. Our findings should be interpreted cautiously, as this report may be underpowered to detect small-to-moderate interaction effects.

Bilateral concordance of left and right HJSCT measurements was quantified using intraclass correlation coefficient (ICC) and assessed using Bland–Altman analysis.

For iterative regression modeling, bilateral mean HJSCT was the primary outcome. We aimed to evaluate whether JIA status was associated with HJSCT measurements after accounting for growth describing factors. Different multivariable linear regression models were fitted with the aim of adjusting for (i) potential non-linearity using different transformation and (ii) relative height, as quantified using a height-for-age z-score (HAZ). Model comparison was based on changes in Akaike Information Criterion (AIC) and likelihood ratio tests for nested models.

Due to the exploratory nature of this study, we also examined interaction effects between JIA status and growth-related factors. To account for heteroscedasticity, models were fitted with robust HC3 standard errors. We calculated 95% confidence intervals (CIs) based on 1000 bootstrap replicates. The effect of JIA subtype was another supplemental analysis.

All tests were two-tailed and *p* value < 0.05 was statistically significant.

## 3. Results

### 3.1. Description of the Study Cohort

We examined measurements of HJSCT using data for 369 pediatric patients, including 187 children with early JIA and 182 controls. Both groups were comparable in age, with medians (IQR) of 9 (5–14) vs. 11 (6–14) years (*p* = 0.079). Sex distribution was also similar (male gender; 36.4% vs. 39.6%, *p* = 0.59). Patients with JIA were characterized by a lower absolute height (mean (SD); 132.9 (28.2) vs. 140.0 (25.5) cm, *p* = 0.013), which is consistent with their younger age distribution. Given the above, and due to strong collinearity between age and height (r = 0.95, VIF > 9), we calculated height-for-age z-scores (thereafter referred to as HAZ) using the WHO Chart Reference (WHO 2006–7 [40,41,42]). Additionally, supplemental analyses with internal sex-specific spline models based on control group data were performed but showed greater instability. Our approach suggests reduced collinearity with VIF below 2.5, with mild age–HAZ correlation (r = −0.13). Importantly, we did not observe significant differences in HAZ scores across the JIA and control groups (mean (SD); 0.07 (1.38) vs. 0.28 (1.09), *p* = 0.11).

### 3.2. Characteristics of Sonographic Measurements

**Figure 2:**
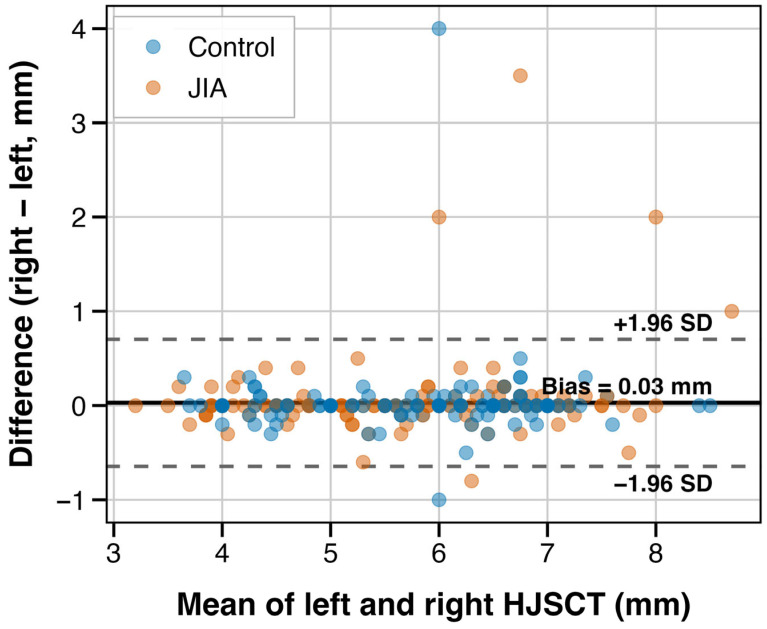
Bland–Altman plot for bilateral HJSCT agreement. Notes: Bland–Altman plot illustrating agreement between left and right hip joint synovial cavity thickness measurements. Solid horizontal line corresponds to mean difference, while dashed lines represent 95% limits of agreement. Orange and blue points correspond to JIA and control group identifiers, respectively.

Left and right HJSCT measurements showed very high bilateral agreement (ICC(2,1) 0.947, 95% CI 0.935–0.956). In Bland–Altman analysis, we also did not observe evidence of systematic bias, with a mean difference of 0.03 mm (95% limits of agreement [LoA] −0.64–0.70 mm; see Figure 2).

### 3.3. Hip Joint Synovial Cavity Thickness in JIA and Controls

**Figure 3:**
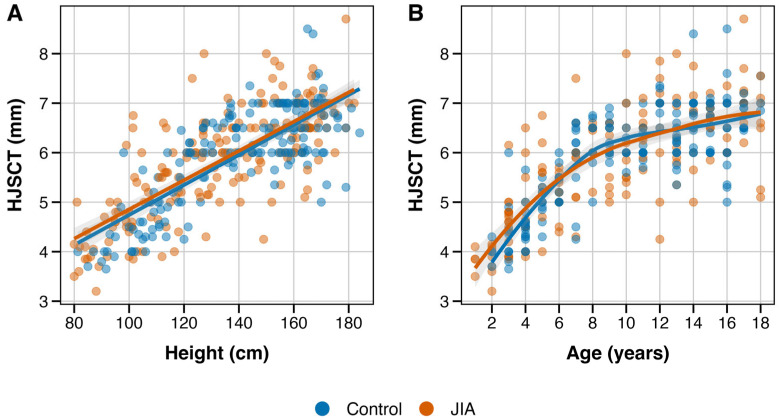
Relationship of HJSCT with patients’ (**A**) height and (**B**) age, stratified by JIA status. Notes: Scatter plot illustrating relationship of hip joint synovial cavity thickness with (**A**) height and (**B**) patients’ age, stratified by JIA status. Smoothed curves are based on linear regression (Panel (**A**)) and LOESS (Panel (**B**)) approaches with standard error-based uncertainty bounds (grey). Of note, LOESS is used for visualization, while formal testing is based uses natural splines (df = 3).

We examined the relationships between anthropometric variables and HJSCT assessments (see Figure 3). HJSCT increased with age in a non-linear pattern, with larger increments during early childhood. HJSCT was also linearly (positively) associated with height. In unadjusted comparison, mean HJSCT was highly comparable between groups (JIA 5.83 (1.09) vs. controls: 5.95 (0.99) mm; difference −0.12 mm, *p* = 0.25). This minimal crude difference (i.e., equivalent to 0.11 SD) illustrates the importance of adjusted analyses to account for the influence of growth-related factors.

Thereafter, we compared several multivariable models with varying functional forms for age and different covariate terms (see Table 2). Based on AIC comparison, we identified Model 7 (natural splines for age with df = 3, with adjustment for HAZ and JIA status; adj. R^2^ = 0.656) as the best. We also tested whether the JIA effect varied with age based on nested model comparison. We did not observe a significant interaction (F(3, 360) 1.01, *p* = 0.39), which suggests that the JIA effect (or lack thereof) may be interpreted as consistent across age groups.

Final model coefficients with HC3 robust standard errors are presented in Table 3. HJSCT showed a non-linear, positive association with age (all spline terms *p* < 0.001). HAZ was recorded with a numerical trend (beta 0.04, 95% CI −0.01–0.09, *p* = 0.11). JIA status was not a significant predictor after covariate adjustment (beta 0.07, 95% CI −0.06–0.19, *p* = 0.30).

Our findings suggest that after accounting for the growth-related factors, HJSCT does not differ significantly between children/adolescents with JIA and the control group. We estimated the JIA effect as 0.07 mm (i.e., equivalent to 0.06 SD), which is clinically negligible.

In supplemental analysis with model bootstrap, we identified Model 7 as the consistently best performer (~80% of 1000 resamples). Of note, patients’ sex was not a significant predictor with addition to the spline model (F(1, 362) 0.00, *p* = 0.99).

### 3.4. Relationship Between JIA Subtype and Hip Joint Synovial Cavity Thickness

**Figure 4:**
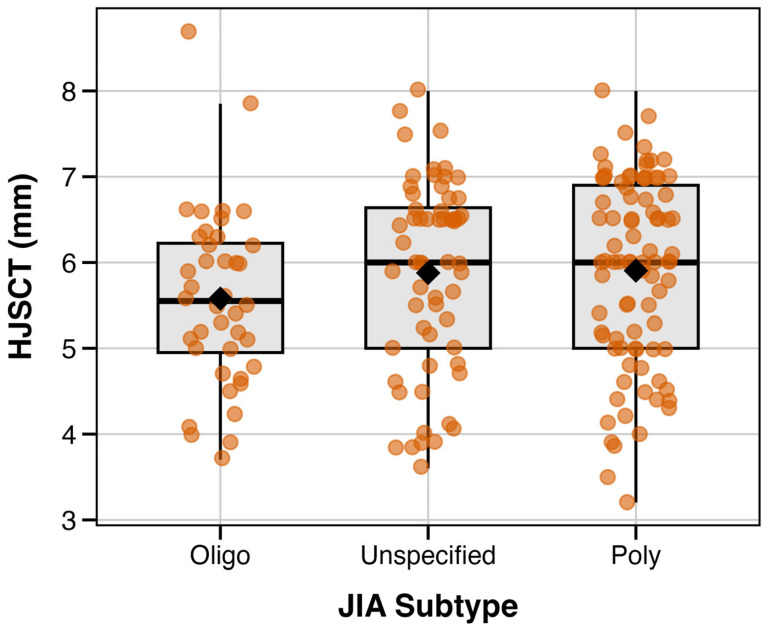
HJSCT distribution by JIA subtype. Notes: Boxes indicate interquartile range with median; diamonds indicate mean values. Individual observations are shown as jittered points.

In additional exploratory analysis, we examined whether HJSCT differed by JIA subtype. Oligoarticular JIA showed numerically lower mean HJSCT, as compared with the polyarticular and undifferentiated subtypes (see Figure 4; Table 4). However, after adjustment for height, JIA subtype was not an independent predictor of HJSCT (*p* = 0.86). Therefore, crude differences are likely to reflect confounding by age (median 7.5 vs. 10 years in oligoarticular vs. other subtypes) rather than actual subtype-specific effects. With the observed sample size and variability, this analysis is likely underpowered due to observed differences of about ~0.3 mm.

### 3.5. Predicting Hip Joint Synovial Cavity Thickness

**Figure 5:**
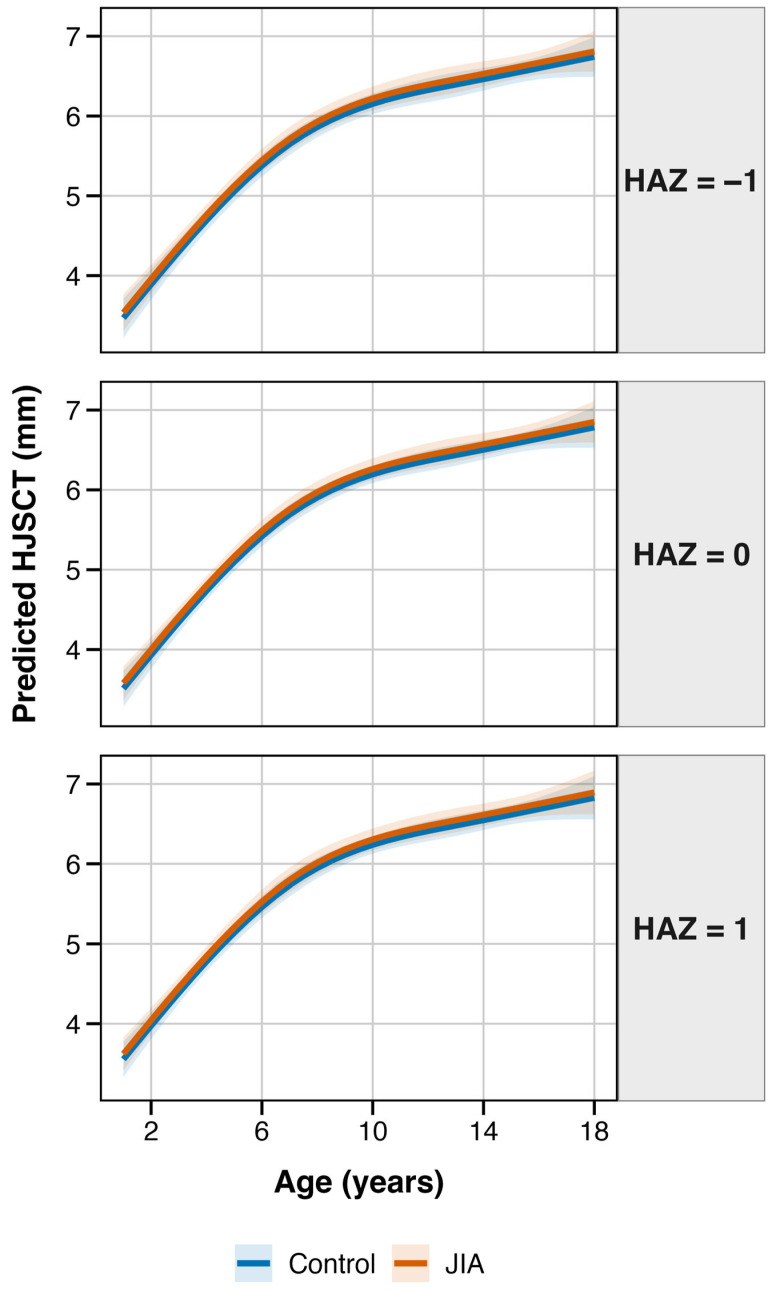
Predicted values of HJSCT by age, stratified by relative height and disease group.

Lastly, we used the final model to derive estimates of age-dependent HJSCT, stratified by HAZ scores (see Figure 5). We observed that HJSCT sharply increases in early childhood (~1.2 mm for 2–5 age group; ~1.1 mm for ages 5–10). As expected, growth rate slowed during adolescence (0.4 mm for ages 10–15; 0.2 mm for ages 15–18). The effects of relative height were additive within the examined age range. Of note, children who were taller-for-age were characterized with ~0.09 mm higher HJSCT assessments than shorter-for-age children (HAZ +1 vs. −1). However, this difference was not statistically significant (*p* = 0.11). We did not observe major differences in the trajectories for JIA and control groups.

## 4. Discussion

This pilot, cross-sectional study evaluated HJSCT in children and adolescents with early JIA, as compared with controls presenting for hip US, but without a relevant, formal diagnosis. The salient finding of this report is that growth-related anthropometric factors are major determinants of HJSCT, accounting for about 65% of the variance in our final model. HJSCT is characterized by a strong, non-linear increase with age. Importantly, in this population without detectable joint effusion, we observed no significant difference in mean bilateral HJSCT between both groups. In exploratory analyses, crude differences across JIA subtypes (i.e., lower HJSCT values in oligoarticular JIA) were attenuated after accounting for growth-related factors. Our findings suggest that potential presence of “subclinical” synovial thickening is not a manifestation of early JIA patients, given the absence of effusion.

Although consensus definitions and protocols are emerging (for example, PIUS-Hip), practice remains heterogeneous and widespread adoption of a single pediatric hip US protocol is still evolving [18,19]. One of the earliest descriptions of painful hip US was first reported by Seltzer in 1980 [43]. While the distance between the femoral surface and the anterior capsule is commonly utilized (on average 4.7 mm) [44,45,46], standardization and subsequent comparison across studies remains difficult. Early protocols measured the posterior capsule margin, but differentiation between capsule layers in patients without effusion is often dependent on echogenicity, presence of fluid and technical factors. To reduce associated bias and maximize practicality, we favored measurement of mean bilateral total thickness (i.e., femur to external capsule margin), which was characterized high concordance and narrow limits of agreement. In the literature, modest variability is observed across different positions/techniques of measurement (common positioning differences are ~1 mm) [39,46].

Our multivariable analysis suggests that HJSCT is a physiological metric tied closely to skeletal maturity. Bone growth is stimulated by a complex interface of genetic, metabolic, and mechanical factors [47,48,49]. Due to strong collinearity between age and raw height (r ~0.95), we used height-for-age z-scores to account for growth status independent of a child’s chronological age. The modest improvement in model fit when including HAZ suggests that sonographic interpretation based solely on “calendar age” may be suboptimal, though this association did not reach statistical significance. Sex differences are less pronounced in childhood, potentially reflecting age-dependent hormonal influences, which may explain the lack of association observed with HJSCT at present [50].

The lack of HJSCT enlargement in the early JIA cohort is important when considering hypotheses of a pre-clinical JIA stage. In rheumatic disorders, systemic inflammation is purported to exert deleterious multiorgan effects due to cytokine spillover from inflamed joints [51]. Elevated acute phase reactants are predictors of unfavorable outcomes in JIA patients [2], including requirement for total hip arthroplasty [6]. While aberrant states of immune activation (e.g., cytokine storm, macrophage activation syndrome) are well studied, the cumulative effects of low-grade inflammation have received less attention. We recruited JIA patients at an early stage of the care pathway, in whom potential overgrowth of synovium could represent an early indicator of disease. We did not detect a difference in HJSCT in early, non-effusive JIA. Therefore, if low-grade inflammation affects capsular thickness, we hypothesize the effect is likely to be small, delayed, or not captured by the HJSCT measurement approach.

In the current study, our exploratory interaction models suggested that associations between HJSCT and growth-related factors do not differ by JIA status. However, given the cross-sectional design and limited clinical characterization, these findings require dedicated study. While US is sensitive for the detection of erosions and effusion [52,53,54], HJSCT is not a standalone marker for subclinical synovitis in early JIA.

Despite the high sensitivity of MRI for detecting early inflammatory changes [55], its cost, sedation requirement and limited accessibility restrict its utility for routine surveillance [56,57]. US is the modality of choice for longitudinal patient evaluation due to practicality. Its use for detection of subclinical synovitis has largely entered into daily practice [53,58] and it is increasingly apparent that a proportion of asymptomatic patients can have imaging abnormalities, which has implications for classification, treatment and outcomes in JIA [58,59]. However, for US to be a reliable screening tool, validation of reference values in different clinical subgroups is important. Our results tentatively support the use of HJSCT reference values for early JIA patients in the absence of effusion.

Importantly, the absence of disease activity indices, inflammatory markers or adjunct MRI data precludes inferential conclusions regarding the potential of inflammation-derived HJSCT variations. It remains to be elucidated whether synovial capsule changes below our assessment threshold can reflect low-grade synovial inflammation.

Bony tissue is difficult to evaluate reliably, which makes US a modality for assessment of soft tissue, fluid presence or localization (e.g., aid in injection or aspiration). Interpretation of musculoskeletal US findings is, however, still evolving. For pediatric patients, there is an unmet need for geographically validated reference values. Defining borderline thresholds for growth and development abnormalities is also of interest but requires in-depth characterization of physiological patterns. For example, cartilage vascularization can appear similar to synovitis on US, while it is a finding that is highly prevalent in healthy subjects [23,60]. Hip joint US enables examination of the femur head surface, which may explain contralateral differences in absolute extremity length (e.g., identifying degenerative changes due to pathological conditions such as slipped capital femoral epiphysis, Perthes disease or septic arthritis). In contrast to effusion, it remains unelucidated whether the thickness of a specific capsular layer could reflect a subclinical state of inflammation.

There are several limitations of this pilot study that need to be acknowledged. The heterogeneity of JIA manifestations is well established, which may obscure arthritis-subtype-specific US characteristics. Our methodology of US assessment with averaging of HJSCT values was undertaken to minimize random error, but it may lead to reduced sensitivity regarding unilateral pathology. While this was a relatively large clinical sample, it is likely underpowered for subgroup analyses, unless the expected effect for JIA subtype is large. Furthermore, the enrollment of early JIA patients with an unspecified symptom-to-diagnosis timeframe carries a high degree of potential bias. The extrapolation of findings is likely relevant only to early JIA patients, in whom synovial tissue exposure to a proinflammatory milieu is brief. The cross-sectional design and lack of detailed clinical and biochemical assessments warrant cautious interpretation and preclude inferential conclusions; accordingly, these observations should inform hypothesis generation rather than immediate clinical decision-making. As a single-center report with an internal protocol, operator- and protocol-specific practices are other sources of bias that could limit generalizability. Prospective longitudinal cohorts tracking HJSCT trajectories alongside validated disease activity measures (e.g., JADAS [61]), treatment response and structural outcomes would be necessary to establish whether serial HJSCT measurements carry prognostic value. Specifically, studies examining whether HJSCT changes precede clinical flares or correlate with inflammatory biomarker trajectories could clarify the predictive utility of capsular assessment beyond effusion detection. The consideration of DMARD and non-steroidal anti-inflammatory treatment effects on sequential HJSCT assessments is also of high value.

## 5. Conclusions

Hip involvement in JIA is a risk factor for poor prognosis, including permanent physical disability. Due to latent and non-specific symptoms, associated risk prediction tools for hip joint involvement remain of high interest. We examined US assessments of hip joint using a cross-sectional sample of 187 early JIA cases and 182 control referrals with negative rheumatic diagnosis. In early JIA patients presenting without effusion, we observed that HJSCT was comparable. Given further validation, our findings support the use of pediatric US for hip assessment based on healthy children reference charts. However, prospective longitudinal studies accounting for disease activity, duration and treatment are highly warranted.

## Figures and Tables

**Table 1:** Differential diagnosis of hip involvement for selected inflammatory and structural conditions.

Condition	Transient Synovitis [30,31,32]	Septic Arthritis [32,33,34,35]	Juvenile Idiopathic Arthritis [26,36]	Avascular Necrosis of the Hip [37]
Age range	2–10 years	<3 years ^$^	Variable *	3–12 years
Localization	Hip, or referred to thigh, knee or abdomen			
Pain	++	+++	+ *	+
Inability to bear weight	+	+	+ *	+
Fever	−/+ (low-grade)	+++	−/+ *	−
Rash	−	−	−/+ *	−
Inflammatory markers (ESR, CRP)	−/+ (mild)	+++	+/++ *	−
Other characteristics	Usually following viral infection; “well child”	“unwell child”; pain with passive range of motion	Often symmetric polyarthritis; variable presentation	Usually unilateral; concurrent risk factors (e.g., steroids, trauma, hemoglobinopathy)
Course	Often self-limited, uncommon recurrence	Acute; rare recurrence	Chronic, may be relapsing–remitting	Chronic, slowly progressive
Treatment	Supportive; oral analgesics	Surgical; IV antibiotics for ~2 weeks (with transition to p.o.)	Analgesics; steroids intra-articularly; DMARDs	Supportive; surgical

Abbreviations: C-reactive protein (CRP), erythrocyte sedimentation rate (ESR), disease-modifying antirheumatic drugs (DMARDs). * Depending on arthritis subtype. ^$^ Can occur at all ages. −, −/+, +, ++, +++ represent a semi-quantitative scale regarding the commonality of manifestations from the Authors perspective.

**Table 2:** Model comparison for HJSCT prediction.

Model	Predictors	Adj. R^2^	AIC Δ
7	ns(Age, 3) + HAZ + JIA	0.656	0
10	ns(Age, 3) + HAZ + JIA + Sex	0.655	2.1
3	log(Age) + HAZ + JIA	0.646	7.9
8	log(Age) + HAZ + JIA + Sex	0.645	9.9
5	log(Age) + JIA	0.643	10.1
6	log(Age) × JIA	0.644	10.2
9	log(Age) + HAZ + JIA × Sex	0.645	11.4
4	(log[Age] + HAZ + JIA)^2^	0.646	11.5
1	Height + JIA	0.595	57.1
2	Height × JIA	0.594	59.0

Notes: Corrected AIC change (Δ) corresponds to difference relative to best model. Abbreviations: AIC, Akaike Information Criterion; JIA, juvenile idiopathic arthritis.

**Table 3:** Summary of final model coefficients for HJSCT (interpretable covariates).

Parameter	Beta	95% CI	*p* Value
Intercept	3.51	3.29–3.72	<0.001
HAZ	0.04	−0.01–0.09	0.11
JIA	0.07	−0.06–0.19	0.30

Notes: 95% confidence intervals (CI) using model-conditional bootstrap; *p* values are based on HC3 robust standard errors. Spline terms for age are omitted for clarity of presentation. Abbreviations: HAZ, height-for-age z-score; JIA, juvenile idiopathic arthritis.

**Table 4:** Comparison of HJSCT by JIA subtype.

Subtype	*n*	Age, Median (Years)	HJSCT, Mean (SD) mm
Oligoarticular	40	7.5	5.58 (1.04)
Polyarticular	87	10	5.91 (1.09)
Undifferentiated	60	10	5.88 (1.13)

## Data Availability

Anonymized data is available from the authors upon reasonable request.

## Appendix A. Proposed Detailed Schematic of Hip Joint Assessment for Pediatric Patients

### Appendix A.1. Anatomical Sites Within the Hip Joint

US assessment of the pediatric hip is based on visualization of several important intra-articular landmarks: (i) the outline of the femur with its ossification nucleus, (ii) the echogenous hyaline cartilage contour that overlies the proximal epiphysis and (iii) the epiphyseal–metaphyseal junction. Additionally, the acetabular rim and the hyperechoic triangular structure corresponding to the labrum should be visualized, most often in the anterosuperior aspect.

### Appendix A.2. Ultrasound Assessment of the Hip Joint

When the patient is in the supine position, the physician places the transducer longitudinally, keeping orientation medial to the anterior inferior iliac spine. It is important to visualize the femoral head as the primary anatomical landmark. Once visualized, at the femur head, the transducer is moved distally until the femur neck junction is in the center. Throughout this process, the angle (position) of the US transducer probe should be optimized to achieve an image of the cortical femoral neck surface (i.e., a linear, hyperechoic structure). Often, the bilaminar structure of the joint capsule is visible, which reflects the anatomical composition of the anterior recess. At this point, the sonographist should evaluate potential distension.

If fluid is visualized, echogenicity alone is not reliable for exudate characterization. However, most often the hyperechoic character may be suggestive of septic arthritis, particularly when combined with increased vascularity of the periarticular soft tissues.

Additional technique considerations include application of pressure on the distal transducer level, while positioning its face parallel to the suspected location of the femoral neck. This position may enable clearer capsule echogenicity assessment but needs to be trained to reduce the risk of compression (e.g., an omission of a small effusion).

The sonographic appearance of the femoral head changes with the child’s development. While between 4 and 5 months of age, the form is mostly cartilaginous, it is possible to visualize small foci of ossification. By 6 months of age, the sonographist should be able to localize ossification centers. With skeletal maturation, the arcuate changes with a hyperechoic arch can be usually identified between 2 and 3 years. In older children, the femur neck appearance is similar to adult structures, though the growth plate leads to a discontinuity of the hyperechoic cortex (between epiphysis and metaphysis).

If joint aspiration is necessary, the physician should identify the anterior recess, as has been described above. We apply the needle along the long axis of the US probe using an inferolateral approach, where the tip is directed toward the femoral head–neck junction.

An alternative approach involves lateral, longitudinal transducer placement over the greater trochanteric region. This view is positioned more cranially than the Graf approach and permits visualization of the joint space superior to the trochanteric outline, facilitating assessment of the labrum and the femoroacetabular articulation.

#### Appendix A.2.1. Soft Tissue Evaluation

We divide the hip into four anatomical regions: anterior, medial, lateral and posterior areas. In pediatric sonography, the apophyses are important landmarks to assess, keeping in mind the patient’s age. We examine the soft tissue using transverse US probe orientation, performing a cranial-to-caudal scan. We should aim for a full-width examination of each anatomical region, with particular attention to the femoral cortical surface.

#### Appendix A.2.2. Anterior Region of the Hip

Examination of this anatomical area is important to visualize two structures: the anterior inferior iliac spine (AIIS), which is the attachment of the rectus femoris tendon (direct head), and the anterior superior iliac spine (ASIS), to which the inguinal ligament, sartorius and tensor fasciae latae are attached. We should assess both structures in both transverse and longitudinal orientations. Depending on the age of the patient, the apophysis can be characterized with different echogenicity (in younger children it is more hypoechoic, which corresponds to cartilaginous structure; in older children, it changes into a smaller, hyperechoic form).

US evaluation of the anterior region is particularly important in case of snapping hip suspicion (dynamic examination). In transverse position at the femoral head level, the iliopsoas tendon is superficial to the joint capsule. When shifting from internal and external rotation positions, abnormal tendon snapping can be visualized.

#### Appendix A.2.3. Medial Area of the Hip

We assess the medial region of the hip in longitudinal position to examine the pubic bone and attachments of adductor muscle. If pain is felt at the lesser trochanter area, the distal attachment of the iliopsoas muscle should be evaluated. We place the US probe longitudinally over the medial thigh with external rotation position of the abducted hip, in modest flexion.

#### Appendix A.2.4. Lateral Area of the Thigh

We place the US probe in transverse position over the greater trochanter. The shifting contour of the cortical surface corresponds to demarcation of anterior and lateral facets. Attachment of the gluteus minimus muscle to the anterior facet should be visualized in the transverse position, thereafter, shifting to the more difficult longitudinal plane (rotation by 90°). Similarly, the gluteus medius tendon can also be assessed.

Examination can be performed in both the supine or lateral decubitus positions (on the contralateral side). When the patient is supine, the sonographist can visualize movement of the iliotibial band and gluteus maximus muscle relative to the trochanter (via rotation and flexion–extension movements of the hip). Ease of transducer gliding is an important finding for the exclusion of abnormalities. Peritrochanteric bursae should also be examined to rule out distension. If the greater trochanter location is characterized by pain, in order to rule out enthesopathy, the insertion of the proximal gluteus medius should be examined.

#### Appendix A.2.5. Posterior Area of the Hip

We examine the posterior hip region primarily to visualize the ischial tuberosity and attachments of the proximal hamstring. When the patient is in the prone position, we place the US probe in a transverse position over the ischial tuberosity and identify the cortical outline of the ischial tuberosity. Anatomically, the tendon of the semitendinosus and biceps femoris is identified superficially, with the deeper placement of the semimembranosus tendon. Thereafter, we rotate the transducer by 90° for longitudinal evaluation. We can also identify the sciatic nerve that locates lateral to the ischial tuberosity and is best seen in the transverse plane—its course can be traced from the exit point at the piriformis, with bifurcation into the tibial and common peroneal nerves within the popliteal fossa.
